# The effect of socioeconomic position in the experience of disability among people with mental disorders: findings from the World Mental Health Survey Initiative Portugal

**DOI:** 10.1186/s12939-018-0821-1

**Published:** 2018-08-07

**Authors:** Ana Antunes, Diana Frasquilho, Sofia Azeredo-Lopes, Manuela Silva, Graça Cardoso, José Miguel Caldas-de-Almeida

**Affiliations:** 1 Chronic Diseases Research Center (CEDOC), Nova Medical School, Nova University of Lisbon, Rua do Instituto Bacteriológico, nº5, 1150-082 Lisbon, Portugal; 2 Nova Medical School, Nova University of Lisbon, Campo Mártires da Pátria 130, 1169-056 Lisbon, Portugal

**Keywords:** Mental disorders, Disability, Socioeconomic position, Social inequalities

## Abstract

**Background:**

Mental disorders are a major cause of disability with impacts on daily functioning and quality of life, which has been associated with socioeconomic disadvantage. The present study aims to assess how socioeconomic position is related to the disability reported by people with mental disorders, using data from the World Mental Health Survey (WMHS) Initiative Portugal.

**Methods:**

Using data from the Portuguese Mental Health Survey, a nationally representative cross-sectional study (*n* = 3849), several logistic regression models with interaction terms were performed to evaluate the effect of different indicators of socioeconomic position on the disability reported by people with any mental disorder (any 12-month mood or anxiety disorder). Odds ratios were estimated at the specific values of the main effects and interaction terms between the presence of any mental disorder and education, employment status, self-perceived financial deprivation and subjective social status.

**Results:**

The prevalence rate of any mood or anxiety disorder was 21.0% (*n* = 788), among which 14.7% (*n* = 115) reported disability. The results show that among people with any 12-month mental disorder, those in the employment category of “retired or others” had two times higher odds of reporting disability (OR = 2.19; 95%CI: 1.06–4.48) when compared to participants categorized as “working”. Likewise, individuals with financial deprivation had two times higher odds of reporting disability when compared to those non-financially deprived (OR = 2.36; 95%CI: 1.31–4.24). The odds ratios obtained for the specific years of education evaluated were not statistically significant but seem to suggest an educational gradient.

**Conclusions:**

The findings of this study indicate that the disability reported by people with mental disorders varies according to socioeconomic position and draw attention to the need to develop policies to address these inequalities.

## Background

Mental disorders are highly prevalent and represent a major cause of disability worldwide [1, 2]. Disability has been defined as functioning restrictions or activity limitations in multiple dimensions of life that results from the interaction between health determinants and contextual factors [3–6]. Mental disorders represent a challenge to individuals’ quality of life, daily functioning and work performance, possibly contributing to reduction of income and standards of living [7–14]. The labour force participation rate of people with mental disorders has been found to be lower when compared to the rest of the population due to higher unemployment rates, sickness absence and early retirement [15–19]. Consequently, studies have shown productivity loss as a main contributor to the economic burden of these disorders [8, 13, 20, 21].

Under a public health perspective, alongside prevention efforts and access to adequate healthcare, it is important to evaluate which factors may contribute to increased levels of disability among people with mental disorders. For instance, studies have suggested a higher risk of disability due to mental disorders among those socioeconomically more disadvantaged [22–26]. Specifically, educational gradients in sickness absence and early retirement due to mental disorders have been found [25, 26], as well as socioeconomic inequalities in onset, duration and recurrence of work related disability due to depression and other mental disorders [22–26]. Moreover, the risk of exclusion from the labour market among people with mental disorders is likely to aggravate existing social inequalities [27].

The identification of socioeconomic inequalities in the experience of disability among people with mental disorders may represent an opportunity to develop interventions to reduce its impact on well-being and associated personal and economic costs [27, 28]. However, the use of different indicators, particularly those assessing disability, limits comparisons across settings. This also represents an important public health challenge in Portugal since the results from the World Mental Health Survey (WMHS) Initiative (2008/9) have shown a high prevalence rate of 12-month mental disorders (22.9%), associated with substantial societal costs, particularly relevant in relation to other countries [8, 29]. Studies using days out of role as an indicator of disability, corresponding to the number of days in the last 30 that individuals were unable to work or carry out their normal activities due to health-related problems, have found mental disorders to be responsible for 20.2% of days out of role in Portugal, in comparison to 16.0% among high-income WMHS countries, assessed through its population attributable risk proportion [8, 13].

This study aimed to examine the role of socioeconomic position in the experience of disability among people with mental disorders. It was hypothesized that the odds of reporting disability vary according to socioeconomic position, affecting disproportionately those more disadvantaged. Socioeconomic position is a concept widely used in epidemiological research and refers to social and economic factors that contribute for an individuals’ position within society [30]. Indicators of socioeconomic position may not be inter-changeable [31] and be differently associated with health outcomes across the life course [30]. Therefore, the indicators included in this study, namely education, employment status, self-reported financial deprivation and subjective social status, were evaluated independently, integrating both the assessment of objective and subjective aspects of socioeconomic position.

Portugal is among the most unequal European countries and an absence of research and policy efforts to effectively tackle health inequalities have been reported [32]. The results of this study may contribute to a better understanding on the effect of social inequalities in the experience of disability among people with mental disorders, for which current knowledge is still scarce, providing valuable insights for policy making.

## Methods

### Design and study sample

Data was collected through a National Mental Health Survey, carried out in Portugal between 2008 and 2009, within the WMHS Initiative, designed to evaluate the prevalence, severity, distribution and consequences of mental disorders through the collection of cross-nationally representative epidemiological data using standardized methods worldwide [2, 33].

A cross-sectional study based on a stratified multistage clustered area probability household sample was administered to a nationally representative sample of respondents. The participants were Portuguese-speaking adults aged 18 years old or above, residing in permanent private dwellings in the country’s mainland. Informed consent was obtained before the interviews and the procedures were approved by the Ethics Committee of the Nova Medical School, Nova University of Lisbon [33].

The survey was conducted by trained lay interviewers on a face-to-face setting, using computer-assisted personal interview (CAPI). The response rate was 57.3%, similar to the surveys in Belgium, France, Germany, and the Netherlands. No substitutions from the initially selected households were allowed when the originally sampled household resident could not be interviewed [33].

In order to reduce respondent burden, internal subsampling was used by dividing the questionnaire in two parts. Part I included the core diagnostic assessment of mental disorders. All respondents meeting the criteria for any mental disorders also completed Part II, together with a probability sample of 25% randomly selected participants who did not meet criteria for any mental disorder. Part II included the assessment of predictors and consequences of mental disorders and use of services [33].

The total number of interviews was 3849. Both modules (Part I and Part II) were administered to 2060 participants. Part I data was weighted to adjust for differential probabilities of selection, between and within households, non-response bias and discrepancies between the sample and the socio-demographic and geographic data distribution from the census population. Part II was additionally weighted to adjust for the differential sampling of Part I participants into Part II. Further details regarding the study design and fieldwork procedures can be found elsewhere [33].

### Measurements

#### 12-month mental disorders

The presence of any mood and anxiety disorder in the 12 months before the interview were evaluated with the version 3.0 of the WHO Composite International Diagnostic Interview (CIDI), a fully-structured diagnostic interview [34]. A clinical reappraisal study compared the diagnoses obtained by the CIDI 3.0 with those generated by the clinician-administered non-patient edition of the Structured Clinical Interview for DSM-IV (SCID) and showed good concordance between the CIDI 3.0 and SCID estimates for 12-month mental disorders [35]. The diagnoses of mental disorders, assessed using the criteria of the American Psychiatric Association’s Diagnostic and Statistical Manual Disorders Fourth Edition (DSM-IV) [36], were the following: anxiety disorders (panic disorder, generalized anxiety disorder, social phobia, specific phobia, agoraphobia without panic disorder, post-traumatic stress disorder, obsessive-compulsive disorder and adult separation anxiety) and mood disorders (major depressive disorder, dysthymia and bipolar disorder including bipolar I and II). A dichotomous variable was created to indicate the presence or absence of any mental disorder in the past year.

#### Disability (WMHS WHODAS-II)

Disability was assessed with the modified version of the World Health Organization Disability Assessment Schedule (WHODAS-II) for the WMHS Initiative (WMHS WHODAS-II). This instrument is based on the International Classification of Functioning, Disability and Health Framework [4] and was applied to the participants of the Part II sample. The internal consistency and validity of the WMHS WHODAS-II has been demonstrated [37]. Difficulties in the 30 days prior to the assessment were evaluated in the following life domains:

1. Understanding and communication (cognitive domain);
2. Moving and getting around (mobility domain);
3. Personal hygiene, dressing, eating and ability to live alone (self-care domain);
4. Interaction with other individuals (social interaction domain);
5. Difficulties carrying out work or normal activities (time out of role domain).

The specific questions included in each domain can be found elsewhere [37]. The domains scores range from 0 to 100, with higher scores meaning greater disability. A global disability score aggregating all domains scores was obtained. Given the distributional properties of the instrument, this score was dichotomized at the 90th percentile to indicate the presence or absence of substantial disability [37].

#### Indicators of socioeconomic position

Education was assessed through the number of years of education reported by the participants, as a continuous variable. Specific years were selected to report the data by choosing the main milestones of the Portuguese educational system (0 years- no education; 4 years- primary education; 9 years- preparatory education; 12- secondary education; 17- university).

Regarding employment status, the participants were classified in the following categories: 1- working at the time of interview or students; 2- unemployed and 3- retired and others (e.g. homemakers and those under sickness absence).

Self-reported financial deprivation was assessed by asking the participants “would you say you have/ your family living here has: 1- more money than you need; 2- just enough for your needs, or 3- not enough to meet your needs”. A dichotomous variable was created considering the participants with or without perceived financial deprivation (more than enough or enough money for needs vs not enough money, respectively).

Subjective social status was measured with the MacArthur scale, which has shown good reliability and validity [38]. The scale consists in a stepladder with rungs numbered from 1 to 10, with the highest value at the top. Participants were asked to consider the ladder as representing the people in Portugal, where those at the top of the ladder would be better off, in contrast to those at the bottom, who have the least money, least education and the least respected jobs or no job. A dichotomous variable characterized the scores into two categories: low or low-mid scores (1–5) and high mid and high scores (6–10) with the first indicating a low subjective social status [38].

#### Covariates

All models were adjusted for age and gender. The presence of any physical disorder was also considered as a covariate since the comorbidity between physical and mental disorders has been associated with higher levels of disability [39]. Physical disorders were assessed with a chronic disorders checklist that has shown good concordance with medical records [40, 41].

### Data analysis

Means, standard deviations (SD), frequencies and percentages were used to describe the population under study. A multivariate logistic regression model was performed to assess the association between disability and presence of any 12-month mental disorder. Multiple logistic regression models included interaction terms to enable the interpretation of the interaction effect of each indicator of socioeconomic position with the presence of any 12-month mental disorder on disability. To be in accordance with the objectives of the study, the odds ratios (OR) were estimated and interpreted at specific levels of the main effects and interaction terms considering the results among individuals with any 12-month mental disorder. Statistical significance was assessed by 95% confidence intervals (95% CI). The standard errors of the odds ratio estimates, used to obtain the confidence intervals, employed values from the variance-covariance matrix of the corresponding model fits [42, 43]. All estimates were weighted according to the characteristics of the study, as previously explained. Data analysis was conducted using Statistical Package for Social Sciences (SPSS) version 22.0 and R version 3.4.2.

## Results

Table 1 presents the demographic, socioeconomic and clinical characteristics of the sample. Of the 3849 participants interviewed, 51.6% (*n* = 2217) were women. The mean age of the participants was 46.38 (SD = 16.88) and the mean years of education were 8.76 (SD = 4.79). The majority of the participants were working at the time of interview (65.1%; *n* = 1362). Financial deprivation was reported by 33.2% (*n* = 732) of the participants and 34.9% (*n* = 1344) perceive themselves to have a low social status in comparison to others in society. The prevalence rate of any 12-month mental disorder was 21.0% (*n* = 788) and the prevalence of disability was 8.6% (*n* = 212). Among people with any mental disorder, higher levels of unemployment (8.3%; *n* = 40), financial deprivation (41.1%; *n* = 303) and low subjective social status (37.6%; *n* = 300) were found. Moreover, 14.7% (*n* = 115) of these participants reported disability.

**Table 1:** Descriptive statistics of the demographic, socioeconomic and clinical characteristics of the WMHS Portugal sample and sub-sample of participants with any mental disorder

	WMHS Portugal total sample (*n* = 3849)	Participants with any mental disorder (*n* = 788)		
Demographic and socioeconomic characteristics	n	%	n	%
Gender^a^				
Female	2217	51.6	596	70.1
Male	1632	48.4	192	29.9
Employment status^b^				
Working	1362	65.1	430	69.6
Unemployed	172	6.8	40	8.3
Retired and others	526	28.1	133	22.2
Financial deprivation^b^				
No	1311	66.8	410	58.9
Yes	732	33.2	303	41.1
Subjective social status^a^				
High	2463	65.1	482	62.4
Low	1344	34.9	300	37.6
	Mean	SD	Mean	SD
Age^a^	46.38	16.88	42.82	15.19
Education^a^	8.76	4.79	9.50	4.64
Clinical characteristics	n	%	n	%
12-month mental disorders^b^				
Any mental disorder	788	21.0	–	–
Physical disorders^b^				
Any physical disorder	1513	68.7	588	82.2
Disability^b^				
Presence of substantial disability	212	8.6	115	14.7

Descriptive statistics of the study sample and sub-sample with any 12-month mental disorder

*SD* standard deviation, *WMHS* World Mental Health Survey

n: unweighted; %, mean, SD: weighted

^a^ Part I weight

^b^ Part II weight

Table 2 shows the association between the presence of any mental disorder and disability. After adjusting for age, gender and presence of any physical disorder, people with any mental disorder were almost 3 times more likely to report disability when compared to those without any mental disorder (OR = 2.82; 95%CI: 1.95–4.09).

**Table 2:** Odds ratio (OR) and respective 95% confidence interval (95%CI) of the association between the presence of any 12-month mental disorder and disability

Any 12-month mental disorder	OR (95% CI)
Yes	2.82 (1.95–4.09) ***
No	Ref.

Part II weight

Model adjusted for age, gender and presence of any physical disorder

*** *p* < 0.001

Table 3 presents the odds ratios of the interaction effects between the presence of any mental disorder and each category of the indicators of socioeconomic position on disability. The results indicate that, after adjusting for age, gender and presence of any physical disorder, the association between disability and presence of any mental disorder varies significantly according to the category of the indicators evaluated, namely employment status (being “retired or others”) and perceived financial deprivation (being financially deprived). Among people with any mental disorder, those classified as “retired or others” were found to be 2.19 times more likely to report disability when compared to those in the working group (OR = 2.19; 95%CI: 1.06–4.48). Likewise, individuals financially deprived were 2.36 times more likely to report disability when compared to those who did not report this situation (OR = 2.36; 95%CI: 1.31–4.24). The same pattern was found regarding unemployment and low subjective social status, although not statistically significant. The results obtained in the specific years of education selected to report data were not statistically significant as well. However, among participants with any mental disorder, those with lower levels of education appeared to be more likely to report disability when compared to the highest level of education and a gradient was suggest by the results (e.g. no education: OR = 1.81, 95%CI: 0.57–5.82; 4 years of education: OR = 1.58, 95%CI: 0.65–3.84; and 12 years of education: OR = 1.19, 95%CI: 0.85–1.68).

**Table 3:** Odds ratios (OR) and respective 95% confidence intervals (95%CI) for disability, considering participants with any 12-month mental disorder, based on the interaction terms with education, employment status, self-perceived financial deprivation and subjective social status

Interaction Effects	OR (95% CI)
Presence of any mental disorder * Education	
No education	1.81 (0.57–5.82)
4 years	1.58 (0.65–3.84)
9 years	1.32 (0.76–2.29)
12 years	1.19 (0.85–1.68)
17 years	Ref.
Presence of any mental disorder * Employment status	
Working or students	Ref.
Unemployed	1.87 (0.78–4.53)
Retired or others	2.19 (1.06–4.48) *
Presence of any mental disorder * Financial deprivation	
No	Ref.
Yes	2.36 (1.31–4.24) *
Presence of any mental disorder * Subjective social status	
High	Ref.
Low	1.45 (0.81, 2.60)

Odds ratio estimates obtained from four multivariate logistic regression models

All models adjusted for gender, age and presence of any physical disorder

Part II weight

* *p* < 0.05

## Discussion

The objective of this study was to evaluate the effect of socioeconomic position on the disability experienced by people with mental disorders. As hypothesised, the findings suggest that the likelihood of reporting disability varies according to socioeconomic position, in particular employment status and perceived financial deprivation. Participants with any mental disorders in the category of “retired or others” and who perceived themselves as financially deprived had two times higher odds of reporting disability, when compared to those working and not financially deprived, respectively. Moreover, despite not reaching statistical significance, an education gradient seems to be suggested by the results, given that among people with any mental disorder, those with the lowest years of education were almost two times more likely to report disability, with the odds decreasing alongside the number of years of education.

The results are in line with previous research. Studies have shown a lower labour force participation of individuals with mental health problems due to early retirement and sickness absence, among other factors [15, 18, 19, 27]. It is important to highlight that in this study early retirement was not evaluated separately but the association was adjusted for age. The findings on perceived financial deprivation are aligned with research suggesting higher levels of economic disadvantage among individuals with disability due to mental disorders [17, 18, 27]. Regarding education, despite the absence of statistical significance, the results are in line with studies that found an education gradient in the risk of early retirement and long term sickness absence due to mental health problems [25, 26]. It has been suggested that individuals with lower socioeconomic position are more likely to have demanding occupations, both physically and psychosocially, or may not have the same opportunities to accommodate their ill-health on their task requirements and working conditions [25]. This may be particularly relevant within the context of the Portuguese welfare system, characterized by providing the smallest public expenditure per capita in social protection in Western Europe, alongside other Southern European countries [44].

The findings of this study should be interpreted within several limitations. The cross-sectional design limits causal inference, namely to understand if the differences in the experience of disability among people with mental disorders according to socioeconomic position are related to factors existing before the onset of disability, to the onset itself or its duration over time [45]. However, two main pathways may operate co-currently: Among people with mental disorders, those with lower socioeconomic position may be more likely to experience disability. Low socioeconomic position has been associated with higher prevalence of mental disorders and disability, due to risk factors such as low educational level, unemployment, precarious working conditions and lower standards of living [18, 27, 28]. Also, the experience of disability among individuals with mental disorders may further aggravate socioeconomic inequalities. The onset and duration of mental disorders and disability have been associated with more adverse economic outcomes such as job loss, reduced income and healthcare expenditure [17, 27, 45].

Furthermore, similar to other research in this area, disability was evaluated in the previous month, whereas mental disorders are 12-month based. For episodic conditions, the past month disability may not include the time period of the disorder, while using a 12-month diagnosis allows the inclusion of remitted disorders that may have residual adverse effects on disability [10, 13]. Besides, the changes made to reduce respondent burden in the WHODAS-II in the WMHS, such as the use of filter questions, impaired the measurement properties with scores having highly skewed distributions with low mean scores and large proportions of zero scores [37]. To address this issue, the cut-off for defining substantial disability (percentile 90th) has been recommended. However, this procedure may mask cross-national differences and caution is needed when comparing the results obtained in this study with those from other countries [37]. Another possible limitation is the use of a broad category of any mental disorder, which does not consider differences that may occur in the experience of disability associated with specific conditions. Finally, this study fails to account for the recent macroeconomic changes in Portugal, one of the European countries most affected by the global financial crisis [46]. Mental health and well-being are likely to deteriorate more immediately and severely than other health outcomes during periods of economic recession [47], contributing to wider health and social inequalities that may not be represented in the results. This scenario may be further aggravated by the absence of effective policies to address social and health inequalities in Portugal [32]. In spite of these limitations, to our knowledge, this study was the first to assess the effect of socioeconomic position in the experience of disability among people with mental disorders in Portugal. Different indicators of socioeconomic position were used, complementing research in this area and drawing attention to the need to conduct longitudinal studies to ascertain the causal pathways involved in these associations. Furthermore, a nationally representative of the Portuguese population and robust instruments to access disability and mental disorders were used. The use of a multi-dimensional instrument to assess disability also represents a major strength of this study since most research uses indicators of productivity loss, which are difficult to compare and only partially assess the experience of disability.

## Conclusions

In conclusion, despite the inability to ascertain the direction of causality, this study establishes the effect of specific social and economic factors in the experience of disability among people with mental disorders. The results highlight the need to further explore how socioeconomic position may contribute to differential patterns of vulnerability among this group and how disability may exacerbate existing social inequalities. Policies aiming to reduce the burden of disability associated to mental disorders may include the promotion of better access to mental health care services, alongside social inclusion and economic measures to protect the rights of people with mental disorders.

## Abbreviations

CI: Confidence interval
CIDI: Composite International Diagnostic Interview
DSM-IV: American Psychiatric Association’s Diagnostic and Statistical Manual Disorders Fourth Edition
OR: Odds ratio
SCID: Structured Clinical Interview for DSM-IV
SD: Standard deviation
WHODAS-II: World Health Organization Disability Assessment Schedule
WMHS: World Mental Health Survey
